# Machine learning and artificial intelligence research for patient benefit: 20 critical questions on transparency, replicability, ethics, and effectiveness

**DOI:** 10.1136/bmj.l6927

**Published:** 2020-03-20

**Authors:** Sebastian Vollmer, Bilal A Mateen, Gergo Bohner, Franz J Király, Rayid Ghani, Pall Jonsson, Sarah Cumbers, Adrian Jonas, Katherine S L McAllister, Puja Myles, David Grainger, Mark Birse, Richard Branson, Karel G M Moons, Gary S Collins, John P A Ioannidis, Chris Holmes, Harry Hemingway

**Affiliations:** 1 Alan Turing Institute, Kings Cross, London, UK; 2 Departments of Mathematics and Statistics, University of Warwick, Coventry, UK; 3 Warwick Medical School, University of Warwick, Coventry, UK; 4 Kings College Hospital, Denmark Hill, London, UK; 5 Department of Statistical Science, University College London, London, UK; 6 University of Chicago, Chicago, IL, USA; 7 Science Policy and Research, National Institute for Health and Care Excellence, Manchester, UK; 8 Health and Social Care Directorate, National Institute for Health and Care Excellence, London, UK; 9 Data and Analytics Group, National Institute for Health and Care Excellence, London, UK; 10 Clinical Practice Research Datalink, Medicines and Healthcare products Regulatory Agency, London, UK; 11 Medicines and Healthcare products Regulatory Agency, London, UK; 12 Julius Centre for Health Sciences and Primary Care, UMC Utrecht, Utrecht University, Utrecht, Netherlands; 13 UK EQUATOR Centre, Centre for Statistics in Medicine, NDORMS, University of Oxford, Oxford, UK; 14 Meta-Research Innovation Centre at Stanford, Stanford University, Stanford, CA, USA; 15 Department of Statistics, University of Oxford, Oxford OX1 3LB, UK; 16 Health Data Research UK London, University College London, London, UK; 17 Institute of Health Informatics, University College London, London, UK; 18 National Institute for Health Research, University College London Hospitals Biomedical Research Centre, University College London, London, UK

## Abstract

Machine learning, artificial intelligence, and other modern statistical methods are providing new opportunities to operationalise previously untapped and rapidly growing sources of data for patient benefit. Despite much promising research currently being undertaken, particularly in imaging, the literature as a whole lacks transparency, clear reporting to facilitate replicability, exploration for potential ethical concerns, and clear demonstrations of effectiveness. Among the many reasons why these problems exist, one of the most important (for which we provide a preliminary solution here) is the current lack of best practice guidance specific to machine learning and artificial intelligence. However, we believe that interdisciplinary groups pursuing research and impact projects involving machine learning and artificial intelligence for health would benefit from explicitly addressing a series of questions concerning transparency, reproducibility, ethics, and effectiveness (TREE). The 20 critical questions proposed here provide a framework for research groups to inform the design, conduct, and reporting; for editors and peer reviewers to evaluate contributions to the literature; and for patients, clinicians and policy makers to critically appraise where new findings may deliver patient benefit.

Machine learning (ML), artificial intelligence (AI), and other modern statistical methods are providing new opportunities to operationalise previously untapped and rapidly growing sources of data for patient benefit. The potential uses include improving diagnostic accuracy,^1^ more reliably predicting prognosis,^2^ targeting treatments,^3^ and increasing the operational efficiency of health systems.^4^ Examples of potentially disruptive technology with early promise include image based diagnostic applications of ML/AI, which have shown the most early clinical promise (eg, deep learning based algorithms improving accuracy in diagnosing retinal pathology compared with that of specialist physicians^5^), or natural language processing used as a tool to extract information from structured and unstructured (that is, free) text embedded in electronic health records.^2^ Although we are only just beginning to understand the wealth of opportunities afforded by these methods, there is growing concern in the academic community that because the products of these methods are not perceived in the same way as other medical (eg, pharmacological) interventions,^6^ they do not have well defined guidelines for development and use and rarely undergo the same degree of scrutiny.

## Need for guidance

Several high profile publications have shown a lack of transparency,^7^ ^8^ replicability,^9^ ethics,^10^ and effectiveness^11^ in the reporting and assessment of ML/AI based prediction models. This growing body of evidence suggests that while many best practice recommendations for design, conduct, analysis, reporting, impact assessment, and clinical implementation can be borrowed from the traditional biostatistics and medical statistics literature,^12^ they are not sufficient to guide the use of ML/AI in research. Producing such guidance is a major undertaking due to the ever-growing battery of ML/AI algorithms and the multifaceted nature of assessing performance and clinical impact. Not taking action is unacceptable, and if we wait for a more definitive solution, we risk wasting valuable work,^13^ ^14^ ^15^ ^16^ ^17^ while allowing futile research to continue unchecked, or worse, translation of ineffective (or even harmful) algorithms from the computer bench to the bedside.

**Summary points**

Clinically relevant research using modern statistical methods (such as machine learning and artificial intelligence) is too often limited by one or more of TREE concerns (transparency, reproducibility, ethics, and effectiveness); addressing these concerns can facilitate appropriate translation from computer bench to patient benefit
Here we propose 20 critical questions that offer a framework for users and generators of ML/AI research
For research generators, the 20 questions can inform the way research groups design, conduct, and report their research
For editors and peer reviewers, the checklist provides a starting point for evaluating the quality and clinical relevance of articles
For the users of such research findings—including healthcare professionals, patients, and the public—the 20 questions highlight important issues for critical appraisal

## Initial framework

We propose a series of 20 critical questions (box 1) to help identify common pitfalls that can undermine ML/AI based applications in health. The questions span issues of transparency, reproducibility, ethics, and effectiveness (TREE). Appendix 1 includes a brief description of how these questions were generated. The questions are not only relevant for those who use the findings (that is, patients and policy makers), but also for those who generate ML/AI health research. We envision this checklist of questions as providing a framework for journal editors, peer reviewers, and those who critically evaluate contributions to the literature; for researchers as a reference to inform the way that research groups design and conduct ML/AI research; for regulators judging algorithm approval; and for educators of clinicians and academic disciplines involved. Current practice in research publication is heterogeneous with relevant questions not clearly dealt with. Clearly further work is needed to build consensus on what constitutes acceptable practice and reporting, but we believe that adoption of this framework as a starting point, and of other related publications,^18^ will help to build trust in the underlying processes and results of health related ML/AI research.

Box 1 **Critical questions for health related technology involving machine learning and artificial intelligence**

**Inception**

1. What is the health question relating to patient benefit?
2. What evidence is there that the development of the algorithm was informed by best practices in clinical research and epidemiological study design?

**Study**

1. When and how should patients be involved in data collection, analysis, deployment, and use?
2. Are the data suitable to answer the clinical question—that is, do they capture the relevant real world heterogeneity, and are they of sufficient detail and quality?
3. Does the validation methodology reflect the real world constraints and operational procedures associated with data collection and storage?
4. What computational and software resources are required for the task, and are the available resources sufficient to tackle this problem?

**Statistical methods**

1. Are the reported performance metrics relevant for the clinical context in which the model will be used?
2. Is the ML/AI algorithm compared to the current best technology, and against other appropriate baselines?
3. Is the reported gain in statistical performance with the ML/AI algorithm justified in the context of any trade-offs?

**Reproducibility**

1. On what basis are data accessible to other researchers?
2. Are the code, software, and all other relevant parts of the prediction modelling pipeline available to others to facilitate replicability?
3. Is there organisational transparency about the flow of data and results?

**Impact evaluation**

1. Are the results generalisable to settings beyond where the system was developed (that is, results reproducibility/external validity)?
2. Does the model create or exacerbate inequities in healthcare by age, sex, ethnicity, or other protected characteristics?
3. What evidence is there that clinicians and patients find the model and its output (reasonably) interpretable?
4. How will evidence of real world model effectiveness in the proposed clinical setting be generated, and how will unintended consequences be prevented?

**Implementation**

1. How is the model being regularly reassessed, and updated as data quality and clinical practice changes (that is, post-deployment monitoring)?
2. Is the ML/AI model cost effective to build, implement, and maintain?
3. How will the potential financial benefits be distributed if the ML/AI model is commercialised?
4. How have the regulatory requirements for accreditation/approval been addressed?

## Critical questions

### Inception (questions 1-2)

#### What is the health question relating to patient benefit?

The vast majority of published clinical prediction models are never used in clinical practice.^19^ One reason for this is the lack of a specific clinical decision making process that the model could meaningfully inform or optimise; simply predicting future events on their own might not help a clinician do anything differently^20^ (in other words: just because we can, it does not mean we should). This is an important departure from the lone wolf attitude, which has helped foster innovation over the past few decades in ML/AI for health. However, it is being increasingly recognised that such research needs to be seen in a wider organisational context to be made most useful. Therefore, we strongly urge researchers embarking on a new project, at the outset, to clarify and state the relevance of their work to healthcare system and patients. In essence, researchers should be cognisant of the path from development to implementation, and be able to describe which parts of the healthcare data science cycle their proposed research engages with. Note that this does not preclude theoretical, proof of concept, or operational research, which either only occupies a small angle of the healthcare data science cycle or only tangentially affects patients (eg, efficiency related gains in an administrative task). What is important, much like the principles on which registration of research is built, is that this expectation is stated up front.

#### What evidence is there that the development of the algorithm was informed by best practices in clinical research and epidemiological study design?

Similar themes to that of historical issues with clinical research are beginning to present in ML/AI based research, such as using outcome variables are predictors, paying little attention to causal pathways, insufficiently detailed descriptions of the conceptualisation of an inception cohort, and documenting exactly what sort of patients made their way into the analysis.^21^ The PECO principles of epidemiological study design (that is, defining a study population, the exposures used, the key comparators, and the clinical outcomes) have an important role in some these issues when they originally arose in health research, and have now become a useful guide for assessments of the quality and relevance of research evidence.^22^ Although developed in the clinical domain, these principles are still highly relevant to ML/AI research, especially in providing a framework on which to ground large scale projects involving electronic health records. This is just one example of how researchers can use clinical frameworks that exist to inform best practice research in the development of ML/AI based projects.

### Study (questions 3-6)

#### When and how should patients be involved in data collection, analysis, deployment, and use?

With the growing use of routinely collected individual participant data (in addition to researcher collected data), often with an alternative legal basis (that is, legitimate interests) to individual consent, it is more important now than ever that patient and public involvement is seen as an adjunct to all research in healthcare, including work related to machine learning. The exemption from seeking individual consent does not mean that the researchers are exempt from engaging patients and public altogether. Thus (where appropriate), healthcare ML/AI projects should include a clear mechanism to evaluate the acceptability of the proposed model and outcomes to those individuals from whom the data was collected, the users (that is, clinicians), and the affected individuals (that is, those for whom the model will be used to inform clinical management).

Several established frameworks^23^ illustrate how patients and the public might be involved in a research project. We would highly encourage researchers to determine which stages of their project, if any, are amenable to patient and public involvement (at inception), for example, identifying the need for a predictive modelling solution, supporting the development of the algorithm (that is, selection of relevant targets, framing of how outcomes are presented), and determining the acceptability of the algorithm in practice. Arguments suggesting that policies pertaining to patient and public involvement should be decided at the political or institutional level does not recognise the agency of individual researchers, and it is for that reason we have included this question, in an effort to reassign the responsibility to those undertaking the work.

#### Are the data suitable to answer the clinical question—that is, do they capture the relevant real world heterogeneity, and are they of sufficient detail and quality?

The key issue here is whether the clinical question can be answered with the data available. For example, a dataset not containing the (known) relevant or important predictors of an outcome is unlikely to satisfactorily answer questions about it. No ML/AI algorithm can produce something from nothing. To help illustrate some of the potential issues involved in determining whether data are of sufficient quality and detail to inform the clinical question of interest, we have briefly described two core areas where researchers frequently have difficulties when attempting to apply ML methods to healthcare related data:

- Intrinsic sample characteristics. If data are available, but are of poor quality or are not relevant, development of a good ML/AI application is unlikely.^24^ The accuracy of data collection methods, sampling of participants, eligibility criteria, and missing data all need to be considered when assessing the potential of developing useful and generalisable ML/AI algorithms.
- Relevance to task. Models are often unable to attain the levels of accuracy seen in training, owing to the likelihood of failure when operating outside the training data range. For example, the decision making system for an image recognition/self-driving car could fail when encountering a cyclist at night for the first time. Hence, the data—including timescale, heterogeneity (differences in data collection such as measuring devices or compliance), population, and situation—should accord with and represent the envisioned clinical application scenario.

#### Does the validation methodology reflect the real world constraints and operational procedures associated with data collection and storage?

Increasingly, ML/AI research is making use of routinely collected data, including healthcare data (eg, electronic health records, clinical imaging, and genomic information), civil administrative data (eg, death records, and educational achievement), and data^25^ from mobile and wearable devices. Information from these sources can arrive in batches, or via a continuous stream, and is often stored in different locations requiring reconciliation, which in and of itself introduces a delay in when specific pieces of data are available for use. In contrast to these real world constraints, ML/AI algorithms are often validated on historical data, yielding performance guaranteed only under the assumption that the data generating process does not change (eg, over time, or across hospitals). In practice, these assumptions are often violated and result in ML/AI models underperforming when deployed in comparison to performance reported during development.^26^

Researchers could consider this problem as two different but related difficulties. The first is the issue of ensuring that a robust validation scheme is developed. For example, methods that take time into account and create temporally disjointed training and test sets^27^ ^28^ might be needed to account for how the data are collected and stored. The second issue is to prevent a useful solution from becoming redundant owing to drift in institutional data collection, or storage methods. However, little can be done by developers and researchers to future proof their work, other than using best practices for reproducibility (that is, clear descriptions of dependencies and modular development of the data ingress pathway, cleaning, pre-processing, and modelling), in order to reduce the amount of work necessary to redeploy a relevant version of the solution.

#### What computational and software resources are required for the task, and are the available resources sufficient to tackle this problem?

Working with millions of parameters is common in many areas of health related prediction modelling, such as image based deep learning^29^ and statistical genetics.^30^ Therefore, it is common practice to ascertain not only the complexity of the data, but also the computational resources available, because these resources can be the limiting factor (much more often than with traditional statistical models) in determining what analyses can be undertaken.^31^ In some situations, more computational resources could in fact allow better models to be trained. For example, without sufficient computer resources, use of models based on complex neural networks could be prohibitively difficult, especially if these large scale models require additional complex operations (eg, regularisation) to prevent overfitting.^32^ ^33^ Ideally, analysis would not be limited by the availability of computational resources, but researchers should understand the constraints within which they are working so that any analysis can be tailored to requirements. Similar problems can arise when using secure computer environments, such as data enclaves or data safe havens, where the relevant software frameworks might not be available and thus would warrant implementation from scratch. Therefore, it is also important to understand the implications of using specific software, because the underlying licence can have far reaching consequences on the commercial potential and other aspects of the algorithm’s future. A brief overview of software licensing for scientist programmers has been published elsewhere.^34^

### Statistical methods (questions 7-9)

#### Are the reported performance metrics relevant for the clinical context in which the model will be used?

The choice of performance metric matters in order to translate good performance in the (training data) evaluation setup to good performance in the eventual clinical setting with patient benefit. This discrepancy in model performance can arise for multiple reasons; the most common of which is that the evaluation metrics are not good proxies for demonstrating improved outcomes for patients (eg, misclassification error for a screening application with imbalanced classes). Another common mistake is choosing a performance metric that is vaguely related to, but not indicative or demonstrative of, improved clinical outcomes for patients. For example, IBM’s Watson For Oncology (WFO)^35^ is an expert system used in several hospitals worldwide to support decision making. However, published works describing WFO do not report relevant statistical (eg, discrimination, calibration) and clinically oriented (eg, net benefit type) performance metrics. Instead, they focus on concordance (true positive rate where the ground truth is provided by physician—that is, the proportion of instances where WFO’s recommendation agrees with that of the treating physician^36^ ^37^ ^38^). We recommend the following guidance for researchers to avoid such pitfalls:

- Consult all relevant parties (eg, patients, data scientists/statisticians, clinicians) to determine the most appropriate formulation of the statistical goal, such as predicting the absolute risk of an event, or establishing a rank ordering or a pattern detection or classification (see question 3).
- Select the appropriate performance metrics. Each goal has its own unique requirements, and making explicit the statistical goal will help researchers ascertain what the relevant measures of predictive performance are for each specific situation. For example, if prediction (not classification) is the goal, then calibration and discrimination are the minimum requirements for reporting. Furthermore, for comparing two models, proper scoring rules should be used (or at least side-by-side histograms). The TRIPOD explanation and elaboration paper provides a reasonable starting point for researcher seeking more information on this issue.^12^
- Report all results. Although training results are unlikely to be sufficient to evidence the usefulness of the model, they provide important insights in the context of the sample characteristics and any out-of-sample results that are also provided. However, unbiased estimates (that is, those that have been adjusted appropriately for overfitting) are the most important to report.

#### Is the ML/AI algorithm compared to the current best technology, and against other appropriate baselines?

ML/AI algorithms should be viewed as health technologies, and at the design stage consideration should be given to identifying the approach that the algorithm might replace. One common way to exaggerate the benefit of ML/AI approaches is to avoid any comparison of ML/AI with null models or the currently used approach and instead compare to sub-par competitors (including inappropriately or weakly developed statistical models), or to avoid a comparison altogether. This “weak comparator” bias has been generally seen in reports of new versus existing prognostic models.^33^ One such example comes from a systematic review of proposed modifications to the Framingham risk score for predicting the risk of a heart attack within 10 years; the review found that most proposed alternatives had flaws in their design, analyses, and reporting that cast doubt on the reliability of the claims for improved prediction.^39^ To simplify this process, we have summarised the three baselines that together form the basis of a robust comparison:

- Model proxies for uninformed guessing, such as predicting the majority class in a classification task. This is the simplest form of sanity check that researchers can use to demonstrate that their ML/AI model is actually learning something. In some instances, probabilistic guessing could be a more appropriate baseline, but the decision of which one to use should be task specific.
- For almost all clinical questions, there will be a standard statistical approach that is well accepted from decades of biostatistics research, for example, proportional hazards models for survival modelling. The impetus is on developers and researchers to show some demonstrable value in using machine learning instead of the standard approach. Recent evidence has shown that these comparisons are often not fair, and favour one set of methods (commonly ML) over classical statistical methods.^40^ We would urge researchers to keep this in mind when carrying out such comparisons.
- The current preferred method standard, whether it is a clinical diagnosis, biochemical test, or pre-existing model. Researchers should show how the model compares to a relevant gold standard. The ML/AI tool does not need to be better than the gold standard, but it is informative to know how the model compares to it. There might be use cases outside of improved accuracy (eg, prediction can be made on a larger class of patients because less data are required). It is the responsibility of the researcher to articulate this in their specific circumstances.

#### Is the reported gain in statistical performance with the ML/AI algorithm justified in the context of any trade-offs?

For a new diagnostic or prognostic tool to be justified for routine use, it must offer a (clinically) meaningful advantage over existing approaches in addressing a specific need,^41^ which requires the use of an appropriate performance metric as discussed previously. Although necessary, the presence of a (clinically) meaningful advantage alone is not sufficient justification, because any improvement must be weighed against the cost of any changes it necessitates (eg, the resource requirement to collect additional data). In a recent paper published by Google, researchers investigated the accuracy of deep learning methods in combination with electronic health records for predicting mortality, readmission, and length of stay.^2^ In the appendix, the paper’s authors compared their deep learning model against a logistic regression model. The area-under-the-curve improvement reported for each of the three tasks ranged from 0.01 to 0.02. If we assume that all caveats pertaining to statistical significance, and the sufficiency of the reported metric for making this next decision are met, is the marginal gain of implementing a complex ML/AI solution worth it, and is the need any more effectively addressed by the deep learning model? Although the answer to that question will certainly be situation specific, it will (at minimum) need to justify the following:

- The cost of developing, deploying, using, and maintaining a deep learning model such as the one described relative to the improvement observed; and
- The need for additional subsidiary models to increase the explainability lost in the transition away from a model with a human interpretable model (eg, with simple coefficients or consisting of a decision tree)

### Reproducibility (questions 10-12)

#### On what basis are data accessible to other researchers?

Data sharing is not an endpoint in itself but rather a means to enhance, verify, and distribute the knowledge generated by the ML/AI algorithm.^42^ Most major funding sources now require applicants to outline a data management and data sharing plan; this can entail (among other things) storing the data in a convenient format along with a data dictionary, a long term archiving plan, and providing an independent access mechanism (eg, a university ethics committee, or a research and development department). Where data used to develop the ML/AI algorithm have been accessed via national data custodians (eg, Clinical Practice Research Datalink,^43^ NHS Digital,^44^ Healthcare Quality Improvement Partnership^45^), clear data access processes have been put in place for independent validation by other researchers. Additionally, data sharing can be undertaken by a wide range of mechanisms, including:

- Making the data available in open repositories such as datadryad.org^46^ (after being anonymised using tools such as Amnesia^47^), or restricted access repositories such as the UK Data Archive^48^;
- Signing data sharing agreements;
- Providing remote access to local computing facilities where the data are stored, as is possible with specific restricted access data enclaves such as NORC at the University of Chicago,^49^ and the electronic Data Research and Innovation Service;^50^
- Open sharing of data modified by privacy-preserving methods.^51^

We acknowledge that free and open sharing of all data are a distant goal, however, our expectation of the near future is that all descriptions of ML/AI algorithm development will be accompanied by clear statements of what tools and mechanism will be used to support access to the data used, for the purposes of replication of reported results. The advent of the facilities described above means that there are fewer reasons to be unable to share data from publicly funded research with other researchers, and as such, we would strongly recommend that investigators establish early on what mechanisms they think are most appropriate and ensure their relevant partners are in agreement.

#### Are the code, software, and all other relevant parts of the prediction modelling pipeline available to others to facilitate replicability^52^?

Reproducibility of research has become a growing concern across many scientific domains,^53^ ^54^ and in the ML/AI field, access to the underlying code and raw data are central to preventing and mitigating reproducibility concerns. A recent example of how concerns regarding reproducibility in medical modelling research have manifested comes from a review of studies published using the Massachusetts Institute of Technology critical care database (MIMIC), which illustrates the degree to which inadequate reporting can affect replication in the prediction modelling.^9^ Specifically, the reproducibility issues that have been identified in the literature occur not only in attempts to recreate reported findings, but also in how authors report data characteristics, such as the inclusion and exclusion criteria used to arrive at the final population of interest. In the review, 28 studies based on the same core dataset (MIMIC) predicting mortality were investigated, and two important results were identified. For more than half of the studies examined, the reproduced sample size differed by more than 25% from the reported sample size because of insufficiently clear descriptions of the inclusion or exclusion criteria. The result of inadequate reporting was that in the replication, the use of off-the-shelf logistic regression and boosted trees on the reproduced samples produced better results in 64% and 82% of the 28 studies, respectively, than the ML/AI model reported in the original study.

These problems could have been easily avoided by providing the project code, specifically the code relating to data cleaning and pre-processing. The RECORD reporting guidelines for studies using routinely collected health data already recommend providing detailed information to this effect,^55^ and several potential solutions can facilitate this process, including code sharing and project curation platforms such GitHub. However, we acknowledge that the ideal level of sharing is not always achievable for many different reasons.^56^ We would highly recommend that, where possible, researchers archive annotated code and include adequate information about software version control to support attempts to reproduce their results.^57^

#### Is there organisational transparency about the flow of data and results?

Patients have strong views about transparency in the flow of data, and how their data are secured.^58^ For patients and their clinicians to trust ML/AI models, they need to understand the interactions that led to the development of the model, whether they are between organisations in the public, not-for-profit and industrial sectors, or within them (eg, transfer from one hospital department to another). Complying with the aforementioned legislative frameworks (eg, the European Union’s General Data Protection Regulation) is necessary, but is not sufficient to show the transparency required to produce trustworthy ML/AI research. The degree of detail needed will differ depending on the institutions involved and the nature of the work being undertaken. Therefore, the responsibility lies with ML/AI algorithm developers and those involved in accessing, transferring, or storing the data, to engage key stakeholders to understand what is required in each particular case. One aspect of the reporting procedure that can help ensure transparency regarding the aforementioned interactions is the inclusion of clear declarations of interest by all involved parties.

### Impact evaluation (questions 13-16)

#### Are the results generalisable to settings beyond where the system was developed (that is, results reproducibility/external validity^59^)?

Even before ML/AI had become established, few validation studies had been done on diagnostic and prognostic tools.^60^ In external validation studies, reductions in the predictive accuracy of models (relative to their original performance in development studies) is expected.^61^ ^62^ Systematic reviews have repeatedly observed this reduced accuracy in the applications of classical statistical models to various healthcare related prediction tasks, from mortality risk prediction in patients with acute kidney injury^63^ to risk prediction of falls in older people.^64^ How this phenomenon is associated with results reproducibility (that is, the production of corroborating results in a new study, having followed the same experimental methods^65^), whether it is a consequence of the inadequate reporting observed in the modelling literature,^59^ or other related issues is unclear. Given the additional complexities introduced by ML/AI algorithms, developers should be proactive in ensuring that sufficient information is provided to allow their models to undergo rigorous but fair^66^ external validation (ideally by independent investigators). This work could include identifying potential datasets for validation experiments at the planning stage, parallel data collection of a validation dataset, or using simulated data to illustrate that the model performs as expected.

#### Does the model create or exacerbate inequities in healthcare by age, sex, ethnicity, or other protected characteristics?

Systematic testing for bias and fairness is the first decision making step in informed model selection, to minimise inequities that could be caused by ML/AI algorithms use.^67^ Although many of the ML/AI algorithms developed will often have bias, it should be compared with the bias in the existing systems being used. One way in which ML/AI algorithms result in bias is by making disproportionate errors in different populations. Depending on how the ML/AI algorithm has been developed (including whether key populations (defined by sex, age, and ethnicity) are sufficiently represented in the data, and included in the training of the algorithm) can influence the predictive accuracy of the algorithm in different subgroups. Thus, when these predictions are used to take actions on individuals, they can create or exacerbate inequities.^68^ The issue of data that are not truly representative of the entire target population is particularly important,^69^ because it highlights the importance of fairness considerations at every point in the project cycle. Other examples of how these issues can manifest in the real world can be found in ProPublica’s analysis of a recidivism prediction tool (the Correctional Offender Management Profiling for Alternative Sanctions software)^10^ and the United States’ diabetes screening criterion,^70^ which both illustrate variation in performance of an algorithm based on race.

The types of performance variation to be investigated depend on the consequent actions (or interventions) that the algorithm is helping to decide between. If the interventions are expensive or have unwanted side-effects, then we would want to minimise disparities in the number of false positive predictions from different subgroups, to prevent unnecessary harm. If the interventions are predominantly assistive, we should be more concerned with disparities in false negatives, to prevent individuals missing out on a potentially beneficial input. The explanation above presupposes that a decision threshold has been set, which might sometimes be outside of a developer’s remit. However, developers still need to demonstrate that when using sensible thresholds, the algorithm does not create or exacerbate inequalities. In fact, several methodological developments in the area of fairness evaluation support this type of analysis,^71^ ^72^ ^73^ and ML/AI developers and health practitioners should engage with these tools. One way in which researchers might demonstrate bias in key subgroups (eg, in minority ethnic groups, or by age) would be to explicitly present these findings so that users of the algorithm know where it has good or poor predictive accuracy.

#### What evidence is there that clinicians and patients find the model and its output (reasonably) interpretable?

Clinical adoption of an algorithm depends on two main factors: its clinical usefulness and its trustworthiness. When the outputs of a prediction model do not directly answer a specific clinical question, its usefulness is limited (as discussed in earlier questions), whereas models whose processing pipeline is difficult to explain and justify to a general audience will invariably limit the trust placed in its outputs,^74^ despite robust and demonstrated statistical gains. However, trust is not the only reason that interpretability in important.^75^ Recent changes in legislation (eg, the EU General Data Protection Regulation) have introduced additional protections for individuals (including a right to an explanation for how a decision was made and where it pertains them^76^), thereby creating a legal requirement to provide insight into the underlying decision making process an algorithm learns. Several partial solutions, including model specific and model agnostic methods (eg, LIME^77^), can be used to claw back interpretability when using ML/AI methods. Legal and moral burdens of explanation to establish trust will vary with the nature of the decision—that is, ML/AI applications in health that influence the allocation of potentially life-prolonging treatments will necessitate a much higher explanatory burden to satisfy those individuals who are affected. Therefore, the sufficiency of any explanations and adequacy of any insight producing method can only be determined through consultation and collaboration with the end users (clinicians), and target audience (patients).

#### How will evidence of real world model effectiveness in the proposed clinical setting be generated, and how will unintended consequences be prevented?

ML/AI tools often carry the misleading aura of self-evident advanced technology, which falsely limits the perceived need for careful validation and verification of their performance, clinical use, and overall use once they begin being used in the routine clinical practice. A recent systematic review showed that only a couple of hundred randomised clinical trials (of a million trials in total) examined how the use of diagnostic tests affected clinical outcomes (and therefore clinical utility).^78^ With regards to the ML/AI domain, Babylon Health’s symptom checker for triage was piloted at a small number of general practices. During early testing, patient focus groups had concerns that there might be “gaming [of] the symptom checker to achieve a GP appointment.”^4^ This example demonstrates how algorithms are not always used as intended in the real world, and that these factors need to be assessed using pragmatic clinical trials.^79^ Early consideration of what the potential pitfalls are of the proposed ML/AL based solution and how it could be manipulated (among other issues) would help researchers develop a better informed framework with which to decide how their tool should be built.

### Implementation (questions 17-20)

#### How is the model being regularly reassessed, and updated as data quality and clinical practice changes (that is, post-deployment monitoring)?

Even if evidence of efficacy and real world effectiveness of a model is sufficient to endorse its widespread use in clinical practice, the effectiveness requires constant review given the dynamic landscape of the healthcare environment. For example, computer aided diagnosis programs have become an integral part of breast cancer screening programmes worldwide since the US Food and Drug Administration (FDA) first approved one for use in 1998,^80^ but are they still as useful as they were 20 years ago? Most of the commercially available tools are based on neural networks which identify regions of interest, and diagnose the identified abnormality (eg, calcification or mass). Early studies showed modest increases in detection rates of breast cancer using computer aided diagnosis or detection (CAD), compared with clinicians working without the aid of a CAD system.^1^ ^81^ However, almost 20 years after the FDA’s first license for a mammography based CAD system, national registry based studies have shown no significant improvement in diagnostic accuracy associated with CAD use in mammography interpretation.^82^ Moreover, researchers have recently demonstrated that incorrect prompts from mammography based CAD systems can actually decrease the sensitivity of more discerning users by up to 0.145 (95% confidence interval 0.034 to 0.257) for difficult diagnoses.^83^ Although the work discussed is not a comprehensive review of CAD in breast cancer, the results suggest the importance for constant re-evaluation of technologies, as their usefulness can change over time. Researchers should aim to plan and develop model performance with the intention to reassess; thus, they need to discuss early on what the necessary mechanisms are to facilitate this process, and how these mechanisms can be integrated at the start of implementation (instead of unplanned additional years later).

#### Is the ML/AI model cost effective to build, implement, and maintain?

Although ML/AI algorithms might offer transformational benefits to healthcare systems and patients, substantial costs can be associated with the development of software, generation and use of data, implementation of a new system in practice, and acting on the additional information provided. Understanding the potential clinical benefit of new models (over and above current practice) alongside the cost or savings introduced by using these models should form part of any healthcare decision maker’s appraisal of ML/AI based technologies. Effective appraisal will require the development of assessment frameworks that take into account both the evidence for effectiveness and the evidence for economic impact. In this area, healthcare decision makers (such as the National Institute for Health and Care Excellence and the FDA) are crucial. They can help developers of ML/AI models by providing clear guidance on the appropriate evidence that should be generated to demonstrate both effectiveness and economic impact, including: credible evidence relating to technical accuracy of the models; the relevant outcomes that show clinical effectiveness in general practice; and, as appropriate, evidence to inform decision makers on the budget impact or the cost effectiveness. Researchers should plan projects with an understanding of how their tool or algorithm will eventually be operationalised.

#### How will the potential financial benefits be distributed if the ML/AI model is commercialised?

Like all technologies, ML/AI algorithms could have a market value. In situations where commercialisation is a goal, health systems and governmental research funding can make a substantial contribution to the creation of an algorithm via the associated unrecoverable costs, such as data acquisition (clinicians’ time, scanners), data annotation (training clinicians who eventually interpret the data generated), and developers’ time (that is, when they are publicly funded researchers). This issue is even more important in a publicly funded health system, because the symbiotic relationship between data-generating institutions and those with the capabilities to create ML/AI algorithms is only possible because of expectation that benefits arising from the data use will be retained (to some degree) by the health system, thereby satisfying the social contract with the public. Therefore, the investment and contributions of a health system or institution to an algorithm should be recognised, and a mechanism to compensate them for having done so should be put in place. Answering this question after the development of an algorithm or ML/AI based tool is often fraught with complexities that can take years to untangle, and thus, we would strongly advise researchers and developers near the end of the planning stages of any project to clarify their institution’s innovation pathway, including the routes to commercialisation and the framework through which this could be achieved.

#### How have the regulatory requirements for accreditation/approval been addressed?

Software products including ML/AI algorithms can be subject to many regulatory requirements, depending on the setting in which the product will be used, from research and development to placing the product on the market (box 2 provides a high level overview of the UK’s regulatory framework). In our experience, while most clinicians are aware of “CE” marking of physical devices (the regulatory framework in the EU and the UK), its application to software products can often be a surprise, which is also true of software developers. Given that the regulatory landscape for health related ML/AI based software has changed substantially over the past decade, and will continue to respond dynamically to innovation for the foreseeable future, discussions regarding the regulatory requirements for products in development should be made early in the planning process of a research project. However, having this conversation once is clearly not sufficient. For example, devices that are developed and used in-house (in the UK) are not currently subject to device regulations, but this will change in 2020 when new updated regulations apply,^87^ ^88^ and as such, regular review of regulatory compliance is necessary.

Box 2 **Overview of the UK’s regulatory framework for health related algorithms involving machine learning (ML) and artificial intelligence (AI)**

Developers should determine whether their ML/AI algorithm falls under the Medical Device Regulations’ remit,^84^ which until 2010 did not regulate independent software products. These regulations cover products that make claims with a medical nature such as: providing diagnostic information, making recommendations for treatment, or providing risk predictions of disease. The Medicines and Healthcare products Regulatory Agency has published guidance for developers that covers this in greater detail.^85^ If an algorithm does fall within the remit of the aforementioned regulation, the developer must then seek regulatory approval or accreditation in the form of a “CE” mark before marketing it. To CE mark an algorithm, the developer must follow one of the applicable conformity assessment routes that, for medium and high risk products, will require the involvement of a notified body to assure the process. The developer must ensure that the device meets the relevant essential requirements before applying the CE mark. These requirements include:

- Benefits to the patient shall outweigh any risks
- Manufacture and design shall take account of the generally acknowledged gold standard
- Devices shall achieve the performance intended by the manufacturer
- Software must be validated according to the gold standard, taking into account the principles of development lifecycle, risk management, validation, and verification
- Confirmation of conformity must be based on clinical data; evaluation of these data must follow a defined and methodologically sound procedure.

In addition to the above, manufacturers are required to have post market surveillance provision to review experience gained from device use and to apply any necessary corrective actions.

Moreover, the use of ML/AI algorithms might be regulated indirectly by other legislation or regulatory agencies. The highest profile additional legislative framework to be aware of might be the European Union’s General Data Protection Regulations, the relevance of which has been discussed elsewhere (questions 3 and 16). In terms of other regulatory agencies who have an important role in the regulation of ML/AI software in health, the United Kingdom’s Care Quality Commission is one group to be aware of, as they are tasked with monitoring compliance with NHS Digital’s Clinical risk management standards^86^; a contractual requirement placed on developers engaging in service provision to the UK’s health service.

## Conclusion: from critical questions to a consensus TREE framework

Similar to how clinicians have been aided by frameworks to evaluate the strength of evidence over previous decades, the ML/AI field should usefully build on what has been learned in traditional statistical approaches for clinical evidence and the quality assurance pipeline.^6^ ^19^ ^89^ ^90^ ^91^ ^92^ ^93^ However, as shown here, some of the challenges are new and different. Encouraging patients, clinicians, academics, and all manner of healthcare decision makers to ask the challenging questions raised here will hopefully contribute to the development of safe and effective ML/AI based tools in healthcare. Developing a definitive framework for how to undertake effective and ethical research in ML/AI will involve many challenges. These challenges include finding common terminology (where key terms partly or fully overlap in meaning), balancing the need for robust empirical evidence of effectiveness without stifling innovation, identifying how best to manage the many open questions regarding best practices of development and communication of results, the role of different venues of communication and reporting, simultaneously providing sufficiently detailed advice to produce actionable guidance for non-experts, and balancing the need for transparency against the risk of undermining intellectual property rights. Addressing these challenges of transparency, reproducibility, ethics, and effectiveness are important in delivering health benefits from ML/AI.

## Web extra

Extra material supplied by authors

Web appendix: Supplementary material

## Notes

Contributors: SV and BAM contributed equally to the manuscript. SV, BAM, and HH conceived the study. BAM, GB, FJK, and SV wrote the first version of the manuscript. The second version of the manuscript, which formed the basis of the submission to *The BMJ*, was written and edited by all the stated authors. All authors read and approved the final and accepted version of the manuscript. The corresponding author attests that all listed authors meet authorship criteria and that no others meeting the criteria have been omitted.

The lead author affirms that the manuscript is an honest, accurate, and transparent account of the work undertaken and being reported; that no important aspects of the work have been purposefully omitted without explanation; and that any discrepancies from the original manuscript as planned have been explained.

Patient and public involvement: No patients were directly involved in the inception of the manuscript, development of the questions, or review of the text before publication.
