# Machine learning and artificial intelligence research for patient benefit: 20 critical questions on transparency, replicability, ethics, and effectiveness

**DOI:** 10.1136/bmj.q2390

**Published:** 2024-10-28

In this paper by Vollmer and colleagues (*BMJ* 2020;368:l6927, doi:10.1136/bmj.l6927, published 20 March 2020), the typeset PDF included an incorrect summary box, which has now been corrected.

