# Supplementary material for: Machine learning and artificial intelligence research for patient benefit: 20 critical questions on transparency, replicability, ethics, and effectiveness
Source: BMJ. 2020 Mar 20;368:l6927. doi: 10.1136/bmj.l6927 (PMC11515850; doi:10.1136/bmj.l6927)
Supplement: Supplementary file 1 — Web appendix: Supplementary material [file vols048975.ww.pdf]

Appendix 1: The Structure and Processes for Achieving Consensus

The aforementioned questions arose as a result of a dialogue between a group of UK-based and international organisations spanning a range of different perspectives, from statutory regulators and national advisory bodies, to academic organisations, including:

- The UK's National Data Science Institute (The Alan Turing Institute [1]), established 2015;
- The UK's Health Data Science at Scale Initiative (HDR UK [2]), established 2018;
- The UK's National Institute for Clinical and Care Excellence (NICE [13]); established in 1999;
- The UK's Medicines and Healthcare products Regulatory Agency (MHRA [4])), established in 2003 (as a merger of the Medicines Control Agency (MCA) and the Medical Devices Agency (MDA));
- The UK's Clinical Practice Research Datalink (CPRD [5]), supported by the Medicines and Healthcare products Regulatory Agency and National Institute of Health Research, is a UK Government research service providing anonymised UK primary care data research since the late 1980s.
- The Enhancing the Quality and Transparency of Health Research (EQUATOR) Network [6], established in 2006;
- The Meta-Research Innovation Centre at Stanford (METRICS) [7], established in 2014;
- The Data Science for Social Good (DSSG) Program [8], established in 2013 and run by the Centre for Data Science and Public Policy at the University of Chicago

There was no formal consensus-reaching process employed to arrive at the submitted manuscript. Instead, discussions regarding the need for such guidance began in a conversation between NICE and the Alan Turing Institute, to which the lead authors [Vollmer, Mateen & Hemmingway] subsequently invited interested parties to contribute over the course of a year. Initial discussions regarding the principles on which standards for ML research might be based identified a series of themes central to high quality health-related research and technologies: transparency, replicability, ethics & effectiveness. Based on these themes, a number of questions were then identified and developed over a series of in-person discussions, conference calls, and collaborative working on a 'online' document, which eventually formed the substance of the manuscript submitted to the BMJ. Following the review process, these questions were then adapted into a more didactic format, with the aim of supporting mid-career researchers about to embark on research that employed machine learning. The pivotal role of the six peer reviewers in arriving at the current formulation of each question cannot be overstated, and readers may like to look at the response to reviewer's letter, where we have collated the itemised responses to the reviewers by question.

References

1) Home [Internet]. The Alan Turing Institute. 2018 [cited 2 December 2018]. Available from: https://www.turing.ac.uk

2) Home - Health Data Research UK [Internet]. Health Data Research UK. 2018 [cited 2 December 2018]. Available from: https://www.hdruk.ac.uk

3) NICE [Internet]. NICE. 2018 [cited 2 December 2018]. Available from: https://www.nice.org.uk

4) Medicines and Healthcare products Regulatory Agency [Internet]. GOV.UK. 2018 [cited 2 December 2018]. Available from: https://www.gov.uk/government/organisations/medicines-and-healthcare-products-regulatory-agency

5) Home [Internet]. Clinical Practice Research Datalink. 2018 [cited 2 December 2018]. Available from: https://www.cprd.com

6) The EQUATOR Network | Enhancing the QUAlity and Transparency Of Health Research [Internet]. Equator-network.org. 2018 [cited 2 December 2018]. Available from: https://www.equator-network.org

7) Meta-Research Innovation Centre at Stanford | METRICS [Internet]. Metrics.stanford.edu. 2018 [cited 2 December 2018]. Available from: https://metrics.stanford.edu

8) Data Science for Social Good [Internet]. Data Science for Social Good. 2018 [cited 2 December 2018]. Available from: https://dssg.uchicago.edu
